# A comprehensive overview of cellular senescence from 1990 to 2021: A machine learning-based bibliometric analysis

**DOI:** 10.3389/fmed.2023.1072359

**Published:** 2023-01-19

**Authors:** Chan Li, Zhaoya Liu, Ruizheng Shi

**Affiliations:** ^1^Department of Geriatrics, The Third Xiangya Hospital, Central South University, Changsha, Hunan, China; ^2^Department of Cardiovascular Medicine, Xiangya Hospital, Central South University, Changsha, Hunan, China

**Keywords:** cellular senescence, bibliometric analysis, LDA analysis, machine learning, MeSH term

## Abstract

**Background:**

As a cellular process, senescence functions to prevent the proliferation of damaged, old and tumor-like cells, as well as participate in embryonic development, tissue repair, etc. This study aimed to analyze the themes and topics of the scientific publications related to cellular senescence in the past three decades by machine learning.

**Methods:**

The MeSH term “cellular senescence” was used for searching publications from 1990 to 2021 on the PubMed database, while the R platform was adopted to obtain associated data. A topic network was constructed by latent Dirichlet allocation (LDA) and the Louvain algorithm.

**Results:**

A total of 21,910 publications were finally recruited in this article. Basic studies (15,382, 70.21%) accounted for the most proportion of publications over the past three decades. Physiology, drug effects, and genetics were the most concerned MeSH terms, while cell proliferation was the leading term since 2010. Three senolytics were indexed by MeSH terms, including quercetin, curcumin, and dasatinib, with the accumulated occurrence of 35, 26, and 22, separately. Three clusters were recognized by LDA and network analyses. Telomere length was the top studied topic in the cluster of physiological function, while cancer cell had been a hot topic in the cluster of pathological function, and protein kinase pathway was the most popular topic in the cluster of molecular mechanism. Notably, the cluster of physiological function showed a poor connection with other clusters.

**Conclusion:**

Cellular senescence has obtained increasing attention over the past three decades. While most of the studies focus on the pathological function and molecular mechanism, more researches should be conducted on the physiological function and the clinical translation of cellular senescence, especially the development and application of senotherapeutics.

## 1. Introduction

Cellular senescence refers to a form of stable cell cycle arrest in response to various stimuli (1). As a cellular process, senescence functions to prevent the proliferation of damaged, old and tumor-like cells, as well as participate in embryonic development, tissue repair, etc. (2, 3). However, cellular senescence is also associated with cancer and age-related diseases, including diabetes, hypertension, Alzheimer’s disease, etc. (4). Due to its significant role in pathophysiological processes, studies related to cellular senescence have dramatically increased in recent years. Nevertheless, the priorities and trends of these publications, which can reflect the research hot spots and suggest future research direction, have not been systematically analyzed and discussed.

Bibliometric analysis is often applied to quantitatively analyze academic literature and provide information about recent research topics and trends in a certain field, such as atrial fibrillation (5), diabetes (6), etc. However, bibliometric analysis on cellular senescence has not been conducted yet, because of the deficiency of effective tools to handle large amounts of literature, and previous bibliometric studies are usually conducted in high-cited articles (7). Natural language processing (NLP), a series of machine learning methods for analyzing human language, has been employed to process medical information in recent years (8). Latent Dirichlet allocation (LDA) is a classic method of NLP, and the application of LDA allows scientists to extract specific themes from a large number of publications (9, 10), which may contribute to the development of bibliometric analysis.

In this study, we analyze the themes and topics of the scientific publications related to cellular senescence in the past three decades by combining the machine learning method LDA with bibliometric analysis. These results may provide an overview of previous researches, reveal hot topics about cellular senescence and, more essentially, provide potential directions for future research.

## 2. Materials and methods

Scientific publications indexed under the medical subject headings (MeSH) term “cellular senescence” from 1990 to 2021 were searched on PubMed at 23 December 2022 (Supplementary material 1). Details of the search results were downloaded in the format of PubMed, and the R package “Bibliometrix” was used to extract metadata including the publication year, the publication type, journal, article abstract, MeSH terms, etc. (11). Publications with “Publication Year” of 2022 or 2023 were excluded for further analyses. The impact factor of journal was determined by journal citation reports 2021. The country/region publication number was determined by the listed countries/regions in each publication, and the thermal world map was created by Gunn Map 2.^1^ MeSH terms with occurrence more than 10 times were included in MeSH analyses. Since this was a bibliometric analysis, ethical approval was not necessary.

Latent Dirichlet allocation, a classic topic modeling method to describe the characteristics of a large number of unstructured texts, was applied to identify the research topics with greater specificity from the abstract of the downloaded articles according to previous studies (12, 13). Using Python, a glossary of feature terms was created by LDA based on how often the vocabulary words coexisted in the abstract of the downloaded articles. Research topics were classified based on these feature terms subsequently, and we set the number of research topics to 50 according to previous research. Then, the two most probable research topics of each article were identified according to how often the feature terms appeared in this article. In addition, the Louvain algorithm was adopted for cluster analysis to conduct a topic network and further reveal the relationship between these topics.

R codes and Python codes used in this article were available on GitHub (14).^2^ Excel and R platforms were applied for the visualization of the work, while the topic network was performed by Gephi^3^ (15).

## 3. Results

### 3.1. Characteristics of the publications

**FIGURE 1:**
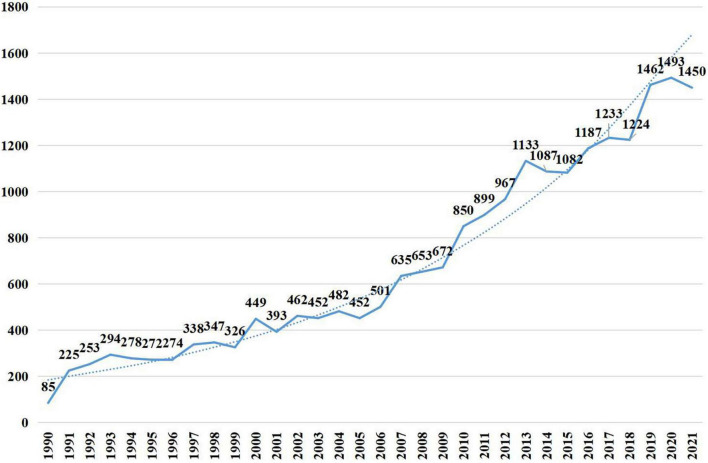
Articles published per year.

**FIGURE 2:**
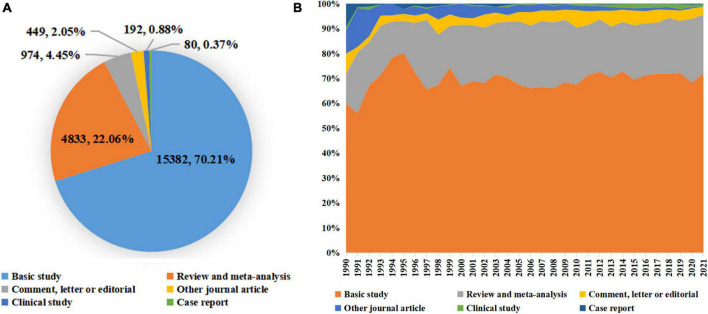
Distribution of publication types. **(A)** The total number and proportion of different publication types on cellular senescence in the past 30 years; **(B)** the distribution of different publication types per year.

**FIGURE 3:**
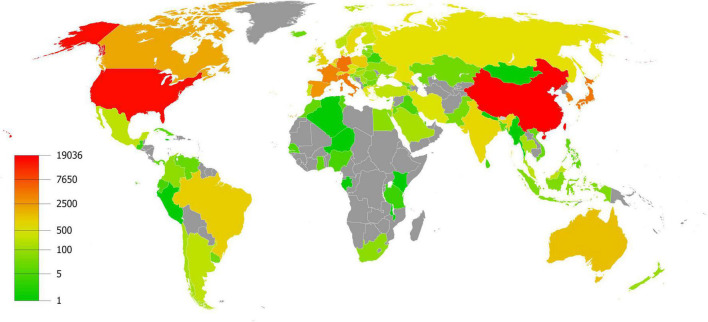
Articles published per country/region.

A total of 21,910 publications indexed under the MeSH term “cellular senescence” were included in our study. On average, 336 articles were published per year from 1990 to 2005. The number of publications continuously increased since 2005 and reached 1,493 per year in 2020 (Figure 1). Overall, the average growth rate from 1990 to 2021 was 9.58%. As shown in Figure 2, the publications were roughly classified into six types, among which basic studies (15,382, 70.21%) accounted for the most proportion of publications over the past three decades. The proportion of review and meta-analysis, comment, letter or editorial, and clinical study slightly increased, while the proportion of case report and other articles declined. In addition, the number of publications in different countries/regions was shown in Figure 3. China, USA, Germany, Japan, and Italy were the top five countries/regions with the largest number of publications. Moreover, the top 10 journals with the most publications were listed in Table 1.

**TABLE 1:** Top 10 journals with most publication related to cellular senescence.

Journal title	Country	Documents	IF 2021
Plos One	USA	464	3.752
Aging (Albany, NY)	USA	463	5.955
Mechanisms of Aging and Development	Switzerland	441	5.498
Aging Cell	UK	431	11.005
Experimental Gerontology	UK	414	4.253
Cell Cycle	USA	319	5.173
International Journal of Molecular Sciences	Switzerland	283	6.208
Scientific Reports	UK	273	4.996
Biochemical and Biophysical Research Communications	USA	263	3.322
Journal of Biological Chemistry	USA	251	5.486

### 3.2. MeSH analyses

For MeSH analyses, 3,256 MeSH terms with a cumulative occurrence of 231, a total of 729 times were included. These terms were divided into three categories: (1) research subject; (2) experimental technology; and (3) others.

The top three MeSH terms of each category were listed in Table 2. Human was the main term of research subject, which was followed by animals and cells. The most common experimental technology was western blot, flow cytometry and transfection. For other MeSH terms, physiology, drug effects and genetics were the most concerned terms. Among which the proportion of drug effects increased most rapidely in the last decade.

**TABLE 2:** Top three medical subject headings (MeSH) terms of each category.

Category	MeSH term	1990–1999		2000–2009		2010–2021	
		Frequency	Proportion (%)	Frequency	Proportion (%)	Frequency	Proportion (%)
Research subject	Humans	1,755	7.06	3,772	6.70	10,904	7.24
	Animals	1,418	5.71	2,562	4.55	6,782	4.50
	Cells, cultured	616	2.48	1,188	2.11	2,191	1.46
Experimental technology	Blotting, western	28	0.11	175	0.31	348	0.23
	Flow cytometry	73	0.29	158	0.28	268	0.18
	Transfection	58	0.23	184	0.33	259	0.17
Others	Cellular senescence/ physiology	623	2.51	1,482	2.63	1,793	1.19
	Cellular senescence/ drug effects	135	0.54	403	0.72	2,031	1.35
	Cellular senescence/ genetics	189	0.76	555	0.99	1,714	1.14

**FIGURE 4:**
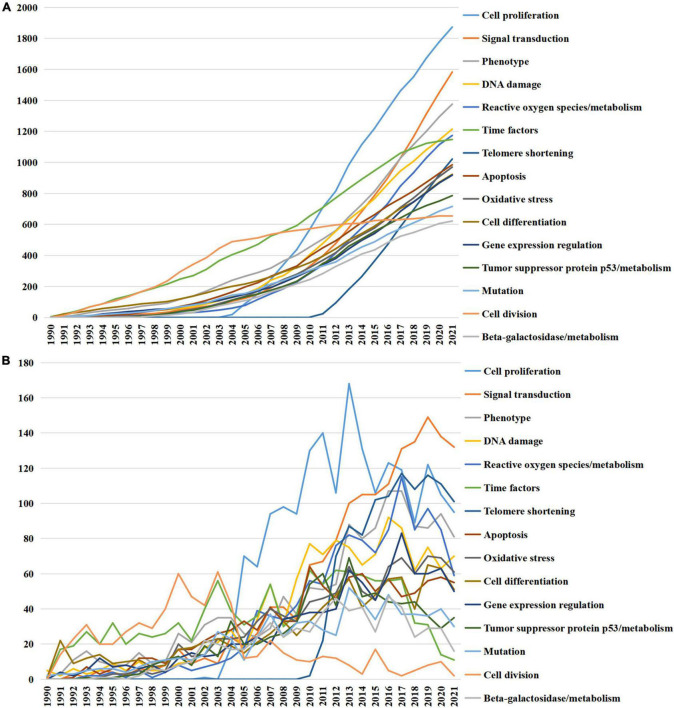
Top 15 medical subject headings (MeSH) terms related to the pathogenesis of cellular senescence. **(A)** Accumulated occurrences; **(B)** annual occurrences.

Furthermore, Figure 4 illustrated the accumulated and annual occurrence of the top 15 MeSH terms related to the pathogenesis of cellular senescence. Cell proliferation and signal transduction were the top two terms over the past three decades and remained popular until 2021. Additionally, the MeSH term telomere shortening appeared in 2010, which increased dramatically over the past decades and had become one of the most focused topics in 2021.

**FIGURE 5:**
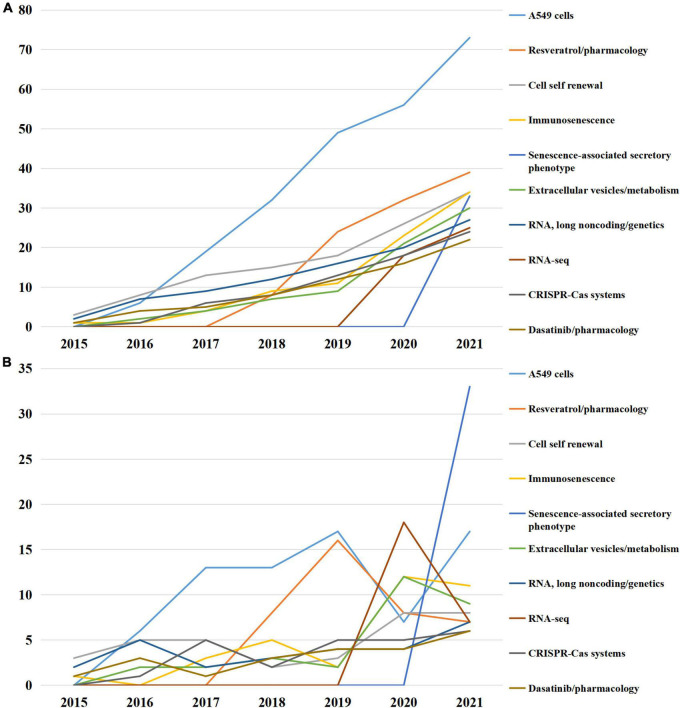
Top 10 medical subject headings (MeSH) terms emerging after 2015. **(A)** Accumulated occurrences; **(B)** annual occurrences.

To further reveal the updated research hot spots, Top 10 MeSH terms emerging after 2015 were emphasized and presented in Figure 5. The conception of cell self renewal, immunosenescence, senescence-associated secretory phenotype (SASP) were proposed, CRISPR-Cas systems and RNA-seq technique were applied, and the roles of resveratrol, dasatinib, long non-coding RNA and extracellular vesicle were investigated.

**FIGURE 6:**
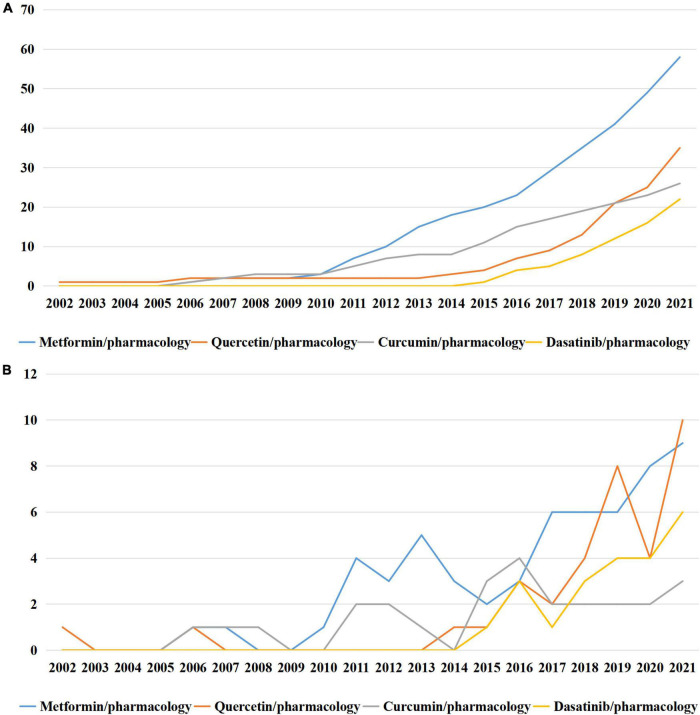
Medical subject headings (MeSH) terms in relate to senolytics and senomorphics. **(A)** Accumulated occurrences; **(B)** annual occurrences.

Since dasatinib, as one of the classic senolytics, had attracted the attention of researchers recently, we searched for other senolytics in the MeSH term list. Currently, only three senolytics were indexed by MeSH terms, including quercetin, curcumin and dasatinib (Figure 6). These senolytics were still not well-studied in the field of cellular senescence, with the accumulated occurrence of 35, 26, and 22, separately. As for senomorphics, the other treatment strategy for senescent cells, the only drug indexed by MeSH terms was metformin, with accumulated occurrence of 58 (Figure 6).

In all, these findings revealed the trend of the hot research fields over the past three decades.

### 3.3. LDA analyses

**FIGURE 7:**
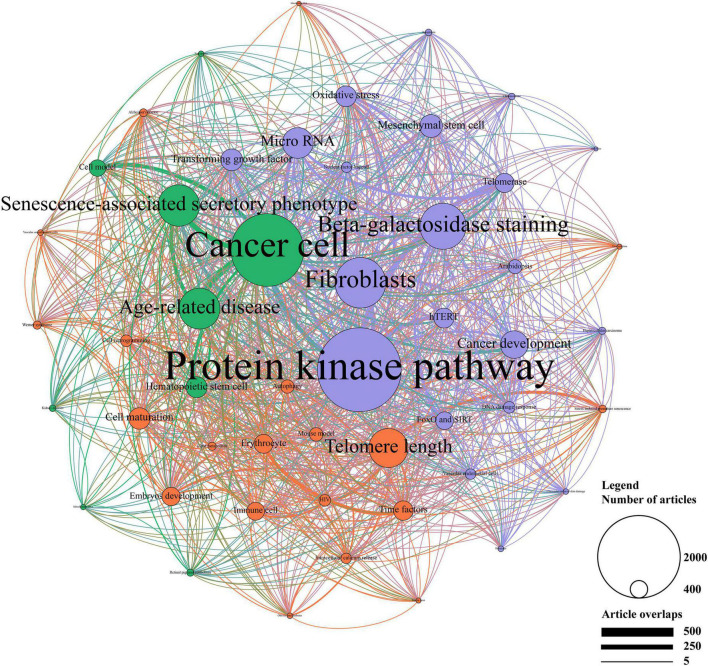
Topic cluster network by latent Dirichlet allocation (LDA). The red cluster represents “physiological function”, the green cluster represents “pathological function”, and the blue cluster represents “molecular mechanism”. The size of the circle represents the number of publications in each topic, while the degree of line thickness represents the weight of connection between topics.

Latent Dirichlet allocation analyses were performed to obtain more detailed research topics from the abstract of the publications and illustrate relationships between prominent topics by creating a network. A total of 20,486 publications were included for LDA analyses except those without an abstract. As shown in Figure 7, three clusters were identified by the Louvain algorithm and presented in different colors, including physiological function (marked in red), pathological function (marked in green), and molecular mechanism (marked in blue).

In the cluster of physiological function, telomere length, cell maturation, time factors, erythrocyte, and embryos development were the top five studied topics. Of these, erythrocyte and time factors showed a strong connection. The cluster of pathological function mainly focused on cancer cell and SASP, followed by age-related disease, hematopoietic stem cell and cell model. Strong correlations were shown between these topics, especially between age-related disease, cancer cell, and SASP. In addition, protein kinase pathway, fibroblasts, beta-galactosidase staining, microRNA, and cancer development accounted for the most important part of the molecular mechanism. Moreover, protein kinase pathway showed a strong connection with fibroblasts and microRNA.

According to the whole network, protein kinase pathway, cancer cell, fibroblasts, beta-galactosidase, and SASP were the primarily focused research topics. Topics in the cluster of physiological function accounted for a relatively small proportion of the network, where more studies should be conducted. Compact connections were shown between the cluster of pathological function and the cluster of molecular mechanism, among which protein kinase pathway and cancer cell showed the strongest association. In the contrast, the cluster of physiological function showed a poor connection with other clusters. Together, LDA analyses revealed the detailed research topics as well as their relationships.

## 4. Discussion

Plenty of studies had been conducted to investigate the roles of cellular senescence since it was discovered in 1961 by Hayflick and Moorhead (16). With increasing attention to this field, plenty of articles reviewed the research progresses of cellular senescence in various diseases, such as cardiovascular diseases (17), endocrine diseases (18), Alzheimer’s disease (19), kidney injury (20), etc. More generally, Amaya-Montoya et al. summarized the basic mechanisms of cellular senescence and the current state of development of therapies against cellular senescence (21), demonstrating a very optimistic opinion in the development of senolytics and senomorphics. However, these comments were conducted based on manual literature acquisition and reading without quantitative analyses, which might influence the objectivity of their opinions on research hot spots. The study, for the first time, quantitatively analyzed 21,910 publications related to cellular senescence during the past three decades by machine learning. Although our research failed to elaborate the detailed mechanisms of cellular senescence and the role of cellular senescence in various diseases, it revealed research hot spots from the perspective of metrology, which may provide potential directions for future research. According to our analyses, the current research situation of senotherapeutics is far from satisfactory, but we are still confident about the future development of senotherapeutics.

The number of studies on cellular senescence increased rapidly over the past three decades. Nearly 2/3 of the investigations were basic studies, while clinical studies only accounted for a small proportion. Though experimental studies may be more appropriate to explore the mechanisms of cellular senescence, their real significance appears when they can be translated into clinical practices. Indeed, some clinical studies have been done on cellular senescence, which promotes the development of this field. The term “senolytics” was created in 2015, and the associated term “senotherapeutics” was added to the MeSH term in 2022, indicating the significance of translational medicine and clinical practice. Thus, more translational and clinical studies should be encouraged in the future.

Medical subject headings analyses were used to figure out the trend of research fields (22). Cell proliferation, as the top theme with rapid risen, is closely correlated to cellular senescence. The arrest of cell proliferation is the characteristic of senescent cells and SASP of senescent cells regulates the proliferation of nearby cells as well (1). The MeSH term telomere shortening was another theme increasing dramatically since it appeared. Telomere shortening caused by cell proliferation is a well-recognized form of replicative senescence (23). It is obvious that these MeSH terms will continue to be research hot spots in the future.

Several hallmarks of senescent cells, including p53, beta-galactosidase and SASP were identified in MeSH analyses. Senescent cells should be defined not by a single trait, but multiple hallmarks. A guide recommend to verify cellular senescence by three different traits, including cell cycle arrest, structural change and additional trait. p53, p16, and p21 are the main markers of cell cycle arrest, while the beta-galactosidase is one of the most widely used markers for structural change. As for additional trait, senescent cells may produce a complex secretome, such as IL-1β, IL-6, and ICAM-1, known as SASP, which is used as a secondary marker to access cellular senescence (24, 25). However, several problems need to be addressed. First, the multiple hallmarks do reflect the complex and heterogeneous mechanisms of cellular senescence, but an universal and robust hallmark is still needed to identify senescence. Furthermore, there are different types of cellular senescence, however, there are only common hallmarks of cellular senescence, indicating the different types of cellular senescence may be hard to identify because of lacking specific hallmarks.

Notably, the significance of senotherapeutics was induced by MeSH analyses. We found that the drug effect of cellular senescence has attracted increasing attention, as shown by the proportion of drug effect in MeSH terms increased from 0.54 to 1.35% during the past three decades. This phenomenon suggested the importance people attached to senotherapeutics, which can be classified into three strategies (senolytics, senomorphics, and senescence-targeting immunotherapeutics). Senolytics selectively killed senescent cells, and senomorphics prevented the progression of young cells to senescent cells by targeting SASPs, while senescence-targeting immunotherapeutics mediated the clearance of senescent cells (26). By searching for novel MeSH terms that did not appear until 2015, we found immunosenescence in the top 10 MeSH terms, indicating that the role of immunity in cellular senescence has been focused by researchers. As we known, the declination of immunological function is closely related to the increasing of senescent cells (27). Strategies targeting senescent immune cells using specific antibodies that recognize senescence surface markers have been applied in anticancer therapies (28). In addition, reversing the abnormal function of senescent immune cells to restore their immune functions is another strategy targeting immune cells, which has also been investigated recently (29). These results indicated that senescence-targeting immunotherapeutics played pivotal roles in delaying the progress of senescence. Meanwhile, the significance of senolytics and senomorphics were also embodied in novel MeSH terms that did not appear until 2015. Dasatinib, as one of the classic senolytics, was added to the MeSH term in 2016, while SASP, as the target of senomorphics, was added to the MeSH term in 2022. Senolytics mainly targeted signal pathways which are activated in senescent cells to kill them (30). Various senolytics, such as quercetin and dasatinib, have been identified and applied to diminish senescent cells in different disease models including myocardial infarction and osteoporosis (31, 32). In contrast to senolytics, senomorphics suppressed the function of SASPs instead of kill senescent cells. Researches showed that cellular senescence is one of the fundamental mechanisms in both age-related and diabetic phenotypes (33). Diabetes can induce cellular senescence and SASP to impair cardiac function independent of age (34). Metformin, as a well-known drug for diabetes, can inhibit SASP by decreasing the expression of IL-1β, IL-6, C-X-C motif chemokine 5 (CXCL5) and NF-kB (35). The function of metformin in aging process has been tested in clinical trials (36), which may become a precursor for future clinical application of senomorphics. It is worth to note that, as we mentioned above, the complexity, various types, and specificity of SASP has not been fully acknowledged. Thus the development of senomorphics may be limited until a comprehensive system is established in the field of SASP. Furthermore, we searched associated senolytics and senomorphics in MeSH terms. Although only three of the senolytics and one senomorphics were added to MeSH terms currently, with relatively small amounts of accumulated occurrence, it did not hinder the fact that researchers have noticed the significance of senotherapeutics in the clinical practice of cellular senescence.

Since the MeSH term was used to catalog publications rather than displaying the detailed content of the publication, plenty of information was ignored in MeSH analyses (37). Therefore, LDA and Louvain algorithm were applied to further investigate the relevant research topics from the abstract of the publications as well as illustrate their relationship. Studies on cellular senescence mainly focused on three fields: physiological function, pathological function and molecular mechanism.

Cellular senescence is usually recognized as a process of damage that leads to multisystem disease or aging. Recently, the physiological function of cellular senescence attracted more attention (2). Telomere length accounted for the predominant proportion in this cluster and was also closely associated with the cluster of pathological function, which was consistent with the result of MeSH analyses. The role of telomere in cellular senescence had been reviewed in a previous article (38). In addition, recent studies highlighted the significant role of cellular senescence in embryo development. Markers and features of senescence had been observed in developing embryo. As the embryo develops, senescent signal changes in a highly organized manner, indicating that senescence in embryo development is a tightly controlled programmed process (39, 40). The specific mechanism of senescence in embryo development remains to be further studied.

Cancer cell, SASP, and age-related disease were the main topics in the cluster of physiological function. The old population is more likely to suffer from chronic diseases, such as diabetes, hypertension, Alzheimer’s disease, etc. The chronic and continuous progress of senescence in cells contributes to the pathogenesis of these diseases mentioned above (41). It is suggested that anti-senescence therapies may be conducive to preventing age-related diseases, which have been discovered and soon become hot research topics (42). However, the safety and efficacy of anti-senescence therapies have not been clearly understood, which demands further clinical studies to validate. It is well-known that cancer is still one of the biggest killers of human, and senescence plays an important role in cancer development and escape (43). Inducing senescence in cancer cells limits the proliferation of cancer cells and may be an optional choice to suppress cancer development. But this therapy may also lead to the relapse of cancer cells from other therapies. The combined use of pro-senescence and senolytic therapies may solve this problem.

Protein kinase pathway was the predominant topic in the molecular mechanism cluster. p38-mitogen-activated protein kinase (MAPK) pathway has been widely investigated in cellular senescence (44). The activation of p38-MAPK activates SASP signal pathway, and compounds targeting p38-MAPK present senolytic function in cancer (23). In addition, microRNAs are reported to promote aging in mouse liver (45). They are associated with other pathways including oxidative stress, p53, telomere shortening, etc. (46). Therefore, microRNAs may be an important linkage between cellular senescence and aging, which need to be further studied.

In conclusion, publications related to cellular senescence increase rapidly during the past 30 years. More research should be focused on the physiological function of cellular senescence and clinical translation, especially the development and application of senotherapeutics.

## Data availability statement

The original contributions presented in this study are included in this article/Supplementary material, further inquiries can be directed to the corresponding authors.

## Author contributions

RS conceived and design of the study. ZL contributed to the design of the study. CL collected and analyzed the data and drafted the manuscript. RS and ZL revised the manuscript. All authors made substantive intellectual contributions to this study to qualify as authors and read and approved the final manuscript.

## Footnotes

1
http://lert.co.nz/map/

2
https://www.jianguoyun.com/p/DUIaWw8Q88SoCxi5xPEEIAA

3
https://gephi.org/

## References

[1] Herranz N Gil J. Mechanisms and functions of cellular senescence. *J Clin Invest.* (2018) 128:1238–46. 10.1172/JCI95148 29608137 PMC5873888

[2] Rhinn M Ritschka B Keyes WM. Cellular senescence in development, regeneration and disease. *Development (Cambridge).* (2019) 146:dev151837. 10.1242/dev.151837 31575608

[3] Calcinotto A Kohli J Zagato E Pellegrini L Demaria M Alimonti A. Cellular senescence: aging, cancer, and injury. *Physiol Rev.* (2019) 99:1047–78. 10.1152/physrev.00020.2018 30648461

[4] Salama R Sadaie M Hoare M Narita M. Cellular senescence and its effector programs. *Genes Dev.* (2014) 28:99–114. 10.1101/gad.235184.113 24449267 PMC3909793

[5] Iftikhar PM Uddin MF Ali F Arastu AH Khan J Munawar M The top most-cited and influential published articles in atrial fibrillation from 1900 to 2019. *Am J Cardiol.* (2020) 125:420–6. 10.1016/j.amjcard.2019.10.053 31785773

[6] Beshyah WS Beshyah SA. Bibliometric analysis of the literature on Ramadan fasting and diabetes in the past three decades (1989-2018). *Diabetes Res Clin Pract.* (2019) 151:313–22. 10.1016/j.diabres.2019.03.023 30904744

[7] Müller AM Ansari P Ebrahim NA Khoo S. Physical activity and aging research: a bibliometric analysis. *J Aging Phys Activ.* (2016) 24:476–83. 10.1123/japa.2015-0188 26671908

[8] Chen X Xie H Wang FL Liu Z Xu J Hao T. A bibliometric analysis of natural language processing in medical research. *BMC Med Inform Decis Mak.* (2018) 18:14. 10.1186/s12911-018-0594-x 29589569 PMC5872501

[9] Gal D Thijs B Glänzel W Sipido KR. Hot topics and trends in cardiovascular research. *Eur Heart J.* (2019) 40:2363–74. 10.1093/eurheartj/ehz282 31162536 PMC6642725

[10] Li C Liu Z Shi R. A bibliometric analysis of 14,822 researches on myocardial reperfusion injury by machine learning. *Int J Environ Res Public Health.* (2021) 18:8231. 10.3390/ijerph18158231 34360526 PMC8345983

[11] Aria M Cuccurullo C. Bibliometrix: an R-tool for comprehensive science mapping analysis. *J Informetr.* (2017) 11:959–75. 10.1016/j.joi.2017.08.007

[12] Wang K Feng C Li M Pei Q Li Y Zhu H A bibliometric analysis of 23,492 publications on rectal cancer by machine learning: basic medical research is needed. *Therap Adv Gastroenterol.* (2020) 13:320843838. 10.1177/1756284820934594 32782478 PMC7385823

[13] Feng C Wu Y Gao L Guo X Wang Z Xing B. Publication landscape analysis on gliomas: how much has been done in the past 25 years? *Front Oncol.* (2019) 9:1463. 10.3389/fonc.2019.01463 32038995 PMC6988829

[14] Dozmorov MG. GitHub statistics as a measure of the impact of open-source bioinformatics software. *Front Bioeng Biotechnol.* (2018) 6:198. 10.3389/fbioe.2018.00198 30619845 PMC6306043

[15] Jacomy M Venturini T Heymann S Bastian M. ForceAtlas2, a continuous graph layout algorithm for handy network visualization designed for the Gephi software. *PLoS One.* (2014) 9:e98679. 10.1371/journal.pone.0098679 24914678 PMC4051631

[16] Hayflick L Moorhead PS. The serial cultivation of human diploid cell strains. *Exp Cell Res.* (1961) 25:585–621. 10.1016/0014-4827(61)90192-6 13905658

[17] Hu C Zhang X Teng T Ma ZG Tang QZ. Cellular senescence in cardiovascular diseases: a systematic review. *Aging Dis.* (2022) 13:103–28. 35111365 10.14336/AD.2021.0927 PMC8782554

[18] Khosla S Farr JN Tchkonia T Kirkland JL. The role of cellular senescence in ageing and endocrine disease. *Nat Rev Endocrinol.* (2020) 16:263–75. 10.1038/s41574-020-0335-y 32161396 PMC7227781

[19] Saez-Atienzar S Masliah E. Cellular senescence and Alzheimer disease: the egg and the chicken scenario. *Nat Rev Neurosci.* (2020) 21:433–44. 10.1038/s41583-020-0325-z 32601397 PMC12548380

[20] Li Y Lerman LO. Cellular senescence: a new player in kidney injury. *Hypertension.* (2020) 76:1069–75. 10.1161/HYPERTENSIONAHA.120.14594 32862712 PMC7484413

[21] Amaya-Montoya M Pérez-Londoño A Guatibonza-García V Vargas-Villanueva A Mendivil CO. Cellular senescence as a therapeutic target for age-related diseases: a review. *Adv Ther.* (2020) 37:1407–24. 10.1007/s12325-020-01287-0 32185730 PMC7140757

[22] Zhao F Shi B Liu R Zhou W Shi D Zhang J. Theme trends and knowledge structure on choroidal neovascularization: a quantitative and co-word analysis. *BMC Ophthalmol.* (2018) 18:86. 10.1186/s12886-018-0752-z 29614994 PMC5883306

[23] Sun Y Coppé J Lam EWF. Cellular senescence: the sought or the unwanted? *Trends Mol Med.* (2018) 24:871–85. 30153969 10.1016/j.molmed.2018.08.002

[24] González-Gualda E Baker AG Fruk L Muñoz-Espín D. A guide to assessing cellular senescence in vitro and in vivo. *FEBS J.* (2021) 288:56–80. 32961620 10.1111/febs.15570

[25] Hernandez-Segura A Nehme J Demaria M. Hallmarks of cellular senescence. *Trends Cell Biol.* (2018) 28:436–53. 29477613 10.1016/j.tcb.2018.02.001

[26] Park J Shin DW. Senotherapeutics and their molecular mechanism for improving aging. *Biomol Ther.* (2022) 30:490–500. 10.4062/biomolther.2022.114 PMC9622307 36226551

[27] Ovadya Y Landsberger T Leins H Vadai E Gal H Biran A Impaired immune surveillance accelerates accumulation of senescent cells and aging. *Nat Commun.* (2018) 9:5435. 10.1038/s41467-018-07825-3 PMC6303397 30575733

[28] Marofi F Motavalli R Safonov VA Thangavelu L Yumashev AV Alexander M CAR T cells in solid tumors: challenges and opportunities. *Stem Cell Res Ther.* (2021) 12:81. 10.1186/s13287-020-02128-1 33494834 PMC7831265

[29] Akbar AN. The convergence of senescence and nutrient sensing during lymphocyte ageing. *Clin Exp Immunol.* (2017) 187:4–5. 10.1111/cei.12876 27690328 PMC5167018

[30] Kirkland JL Tchkonia T. Cellular senescence: a translational perspective. *EBioMedicine.* (2017) 21:21–8. 10.1016/j.ebiom.2017.04.013 28416161 PMC5514381

[31] Zhu Y Tchkonia T Pirtskhalava T Gower AC Ding H Giorgadze N The Achilles’ heel of senescent cells: from transcriptome to senolytic drugs. *Aging Cell.* (2015) 14:644–58. 10.1111/acel.12344 25754370 PMC4531078

[32] Salerno N Marino F Scalise M Salerno L Molinaro C Filardo A Pharmacological clearance of senescent cells improves cardiac remodeling and function after myocardial infarction in female aged mice. *Mech Ageing Dev.* (2022) 208:111740. 10.1016/j.mad.2022.111740 36150603

[33] Palmer AK Gustafson B Kirkland JL Smith U. Cellular senescence: at the nexus between ageing and diabetes. *Diabetologia.* (2019) 62:1835–41. 10.1007/s00125-019-4934-x 31451866 PMC6731336

[34] Marino F Scalise M Salerno N Salerno L Molinaro C Cappetta D Diabetes-induced cellular senescence and senescence-associated secretory phenotype impair cardiac regeneration and function independently of age. *Diabetes.* (2022) 71:1081–98. 10.2337/db21-0536 35108360 PMC9490451

[35] Moiseeva O Deschênes-Simard X St-Germain E Igelmann S Huot G Cadar AE Metformin inhibits the senescence-associated secretory phenotype by interfering with IKK/NF-κB activation. *Aging Cell.* (2013) 12:489–98. 10.1111/acel.12075 23521863

[36] Kulkarni AS Gubbi S Barzilai N. Benefits of metformin in attenuating the hallmarks of aging. *Cell Metab.* (2020) 32:15–30. 10.1016/j.cmet.2020.04.001 32333835 PMC7347426

[37] Nelson SJ Schulman J. Orthopaedic literature and MeSH. *Clin Orthop Relat Res.* (2010) 468:2621–6. 10.1007/s11999-010-1387-4 20623263 PMC3049625

[38] Liu J Wang L Wang Z Liu J. Roles of telomere biology in cell senescence, replicative and chronological ageing. *Cells (Basel, Switzerland).* (2019) 8:54. 10.3390/cells8010054 PMC6356700 30650660

[39] Munoz-Espin D Canamero M Maraver A Gomez-Lopez G Contreras J Murillo-Cuesta S Programmed cell senescence during mammalian embryonic development. *Cell.* (2013) 155:1104–18. 10.1016/j.cell.2013.10.019 24238962

[40] Storer M Mas A Robert-Moreno A Pecoraro M Ortells MC Di Giacomo V Senescence is a developmental mechanism that contributes to embryonic growth and patterning. *Cell.* (2013) 155:1119–30. 10.1016/j.cell.2013.10.041 24238961

[41] Childs BG Durik M Baker DJ Deursen JMAV. Cellular senescence in aging and age-related disease: from mechanisms to therapy. *Nat Med.* (2015) 21:1424–35. 10.1038/nm.4000 26646499 PMC4748967

[42] Gurãu F Baldoni S Prattichizzo F Espinosa E Amenta F Procopio AD Anti-senescence compounds: a potential nutraceutical approach to healthy aging. *Ageing Res Rev.* (2018) 46:14–31. 10.1016/j.arr.2018.05.001 29742452

[43] Perez-Mancera PA Young AR Narita M. Inside and out: the activities of senescence in cancer. *Nat Rev Cancer.* (2014) 14:547–58. 25030953 10.1038/nrc3773

[44] Xu Y Li N Xiang R Sun P. Emerging roles of the p38 MAPK and PI3K/AKT/mTOR pathways in oncogene-induced senescence. *Trends Biochem Sci (Amsterdam Regular Ed).* (2014) 39:268–76. 10.1016/j.tibs.2014.04.004 24818748 PMC4358807

[45] Maes OC An J Sarojini H Wang E. Murine microRNAs implicated in liver functions and aging process. *Mech Ageing Dev.* (2008) 129:534–41. 10.1016/j.mad.2008.05.004 18561983

[46] Reddy PH Williams J Smith F Bhatti JS Kumar S Vijayan M MicroRNAs, aging, cellular senescence, and Alzheimer’s disease. *Prog Mol Biol Transl.* (2017) 146:127–71. 10.1016/bs.pmbts.2016.12.009 28253983

